# Primary Cutaneous Melanoma Arising in a Long-Standing Irradiated Keloid

**DOI:** 10.1155/2012/165319

**Published:** 2012-02-07

**Authors:** Lindsay M. Fish, Lisa Duncan, Keith D. Gray, John L. Bell, James M. Lewis

**Affiliations:** ^1^Department of Surgery, University of Tennessee Graduate School of Medicine, Knoxville, TN 37920, USA; ^2^Department of Pathology, University of Tennessee Graduate School of Medicine, Knoxville, TN 37920, USA

## Abstract

Ionizing radiation has been used therapeutically for a variety of clinical conditions, including treatment of hypertrophic keloids. Keloids may rarely be associated with malignancy, but the use of low-dose ionizing radiation is associated with an increased risk of cutaneous malignancies. We describe a case in which a primary desmoplastic melanoma arose in a long-standing, previously irradiated keloid.

## 1. Introduction

Historically, ionizing radiation has been used to treat a variety of clinical conditions including tinea capitis and for treatment of prominent keloids [1]. Over time, however, people who have been treated with radiation for cutaneous lesions have been shown to be at an increased risk of developing cutaneous malignancy in the treated field.

## 2. Case Presentation

A 57-year-old man with an extensive life-time history of sun exposure and long-term smoking was evaluated for a recurrent cutaneous chest wall keloid. The keloid initially developed secondary to a varicella zoster infection as a child. At age 9, the keloid was treated with low-dose external beam irradiation and essentially resolved. Many years later and over a period of eighteen months prior to clinician reevaluation in 2009, the lesion began to grow in size. Corticosteroid injections failed to induce regression and a punch biopsy was performed.

Pathology from the punch biopsy revealed a primary desmoplastic melanoma, at least 5.0 mm thick with positive margins arising within a keloid. The patient's review of systems revealed vague muscle and rib pains, right-sided headaches, abdominal pain, and a forty-five-pound weight loss. Staging workup was completed to investigate these symptoms and revealed no evidence of metastatic disease.

**Figure 1:**
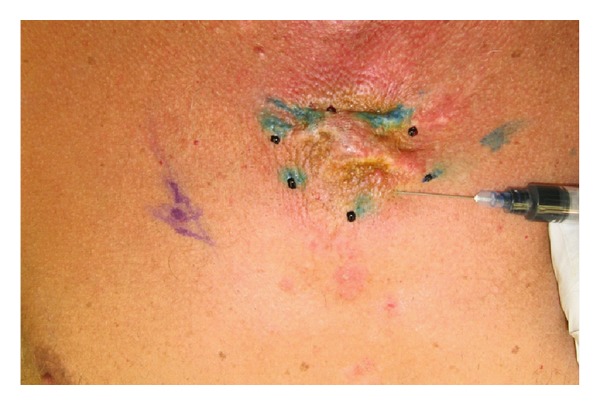
Keloid appearance at presentation. (methylene blue injection for sentinel lymph node biopsy).

**Figure 2:**
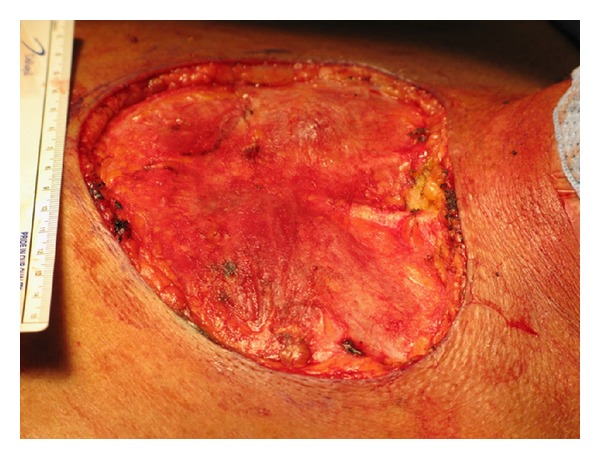
Surgical defect measuring 145 cm^2^.

**Figure 3:**
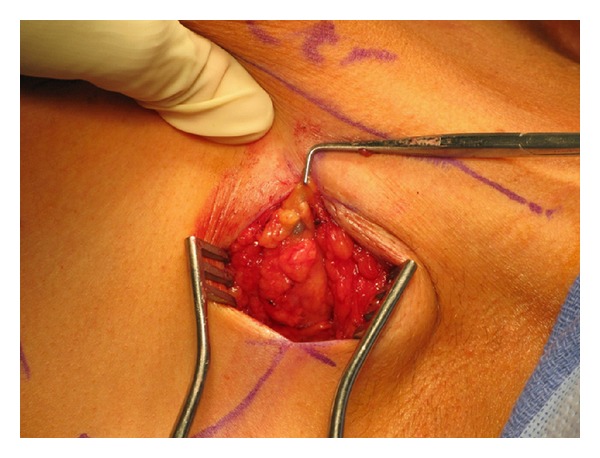
Axillary sentinel lymph node biopsy.

Clinically, it was impossible to differentiate keloid from desmoplastic melanoma. Thus, a two-stage surgical procedure was performed with initial resection of the primary site including the entire keloid with 2 cm margins and bilateral axillary sentinel node biopsy (Figures 1, 2, and 3). Temporary coverage of the primary resection defect was performed with porcine dermal substitute (not pictured) to ensure a margin free resection prior to reconstruction, since it was difficult to determine how deep the melanoma had invaded macroscopically.

**Figure 4:**
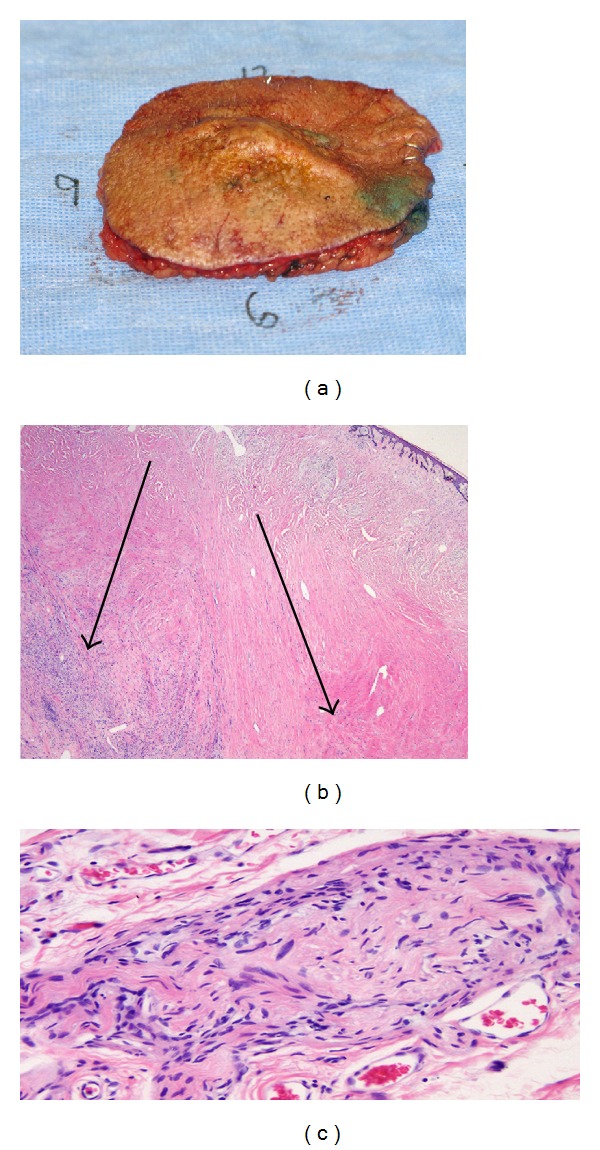
(a) Surgical specimen for pathology. (b) Desmoplastic melanoma (left arrow) and dermal scar (right arrow). (c) High-power view of desmoplastic melanoma.

**Figure 5:**
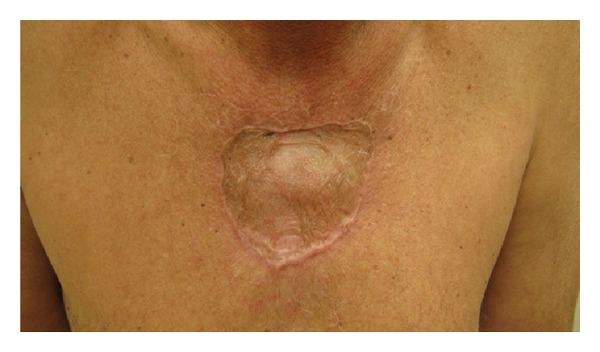
Partial-thickness skin graft after complete healing.

Final pathology revealed desmoplastic melanoma occurring in the keloid with clear radial margins and a deep margin within 0.1 cm of the resection (Figures 4(a), 4(b), and 4(c)). Thus, one week after the initial surgery, the patient returned to the operating room and a new deep margin was obtained, which was negative for malignancy. Bilateral sentinel lymph nodes were negative for metastasis. Partial thickness skin graft reconstruction was then performed to cover the previously created 145 cm^2^ defect. The partial thickness skin graft had a 100% take (Figure 5). The patient was offered adjuvant immunotherapy but declined. He remained recurrence free from melanoma at his thirteen month follow-up evaluation. However, two months after this follow-up appointment, he was diagnosed with a primary squamous cell carcinoma of the lung, which rapidly progressed causing his death, just three months later.

## 3. Discussion

Ionizing radiation has been used to treat a variety of clinical conditions including tinea capitis and prominent keloids. The use of radiation to treat benign conditions is controversial and is associated with a 10% increased risk of subsequent development of cutaneous malignancy [1]. As early as 1902, there have been case reports and hospital series of radiation-induced skin cancer [2]. Shore and colleagues studied radiation-induced skin malignancies in 2,200 children irradiated for tinea capitis compared to 1,400 children treated with other modalities. They report a relative risk of 3.8 and absolute risk of 2.3/10^4^ persons per year per Gy at an average skin dose of 4.5 Gy [3]. In this study the association of cutaneous malignancy was only seen in Caucasians, suggesting a relationship with ultraviolet radiation and skin pigment. Ron et al. reported on over 10,000 patients irradiated for tinea capitis compared to a control group. They found 44 cutaneous malignancies in the treatment group compared to 16 in the control group. There were a total of 2 melanomas in the treatment group out of 44, compared to 1 melanoma in 16 malignancies in the control group. Benign skin tumors were also more common in the irradiated cohort [4].

The risk of developing malignancy after irradiating keloids is not well documented and is limited to mostly case reports. Botwood et al. documented bilateral breast malignancies developing in a 57-year-old woman 35 years following irradiation treatments for keloids that developed after burns to her torso [5]. Sainsbury et al. recently documented a keloid that became involved with CLL after multiple operations, steroid injections, and irradiation [6]. There are no large series documenting the increased risk of cutaneous malignancy in patients with keloids treated with ionizing radiation. Klumpar et al. reported treatment of 126 keloids with surgical excision followed by radiation and a median followup of 12 years. In their patient population with followup for as long as 17 years, they reported no evidence of dysplastic changes or malignancy related to the excision and radiation [7].

While most cutaneous malignancies associated with ionizing radiation are basal cell carcinomas, there are a few documented cases of melanoma arising in an irradiated cutaneous field. Maalej et al. documented 150 cases of radio-induced malignancies of scalp malignancies as a result of irradiation for tinea capitis, which was a common practice in the 1950s. Only four of these cases were malignant melanoma [8]. Fink and Bates documented an elevated relative risk for melanoma after occupational, environmental, and medical exposure to external ionizing radiation in several studies. Relative risks for leukemia in each of these studies were used to confirm the likelihood of exposure to ionizing radiation. In general, exposure categories with elevated risks of leukemia had an elevated risk for melanoma [9]. Trefzer et al. reported two patients that developed melanoma or melanoma metastases within a radiated field, six and forty-three years after radiation therapy [10]. Similarly, Miracco et al. reported two cases of unusual neoplasms secondary to radiation therapy. One was a pleomorphic rhabdomyosarcoma and the other a lentigoid malignant melanoma [11]. Cancers secondary to radiation with a long latency period are not uncommon; however, the development of melanoma within them is extremely rare.

Neoplasms have rarely been reported arising in the setting of a keloid. Theopold et al. documented a single case of a malignant blue nevus arising on the earlobe of an Afro-Caribbean man, who had a keloid scar removed from the same site just eight years prior to his presentation [12]. In 1978, Hiss and Shafir described a case of pseudomelanoma or benign melanocytic nevus that occurs after nevus removal and resembles superficial spreading melanoma. The lesion developed in a keloid after a shave biopsy was performed six months earlier [13]. To our knowledge there has been no description of malignant melanoma arising in a keloid; however, the above cases highlight the importance of histological analysis of all suspicious or unusual appearing lesions.

 The subtype of our patient's melanoma was desmoplastic. Desmoplastic melanoma (DM) is a rare, fibrosing, spindle-cell variant of melanoma. They are often misdiagnosed as a scar or keloid, leading to late detection and a worse outcome for the patient [14, 15]. They are frequently seen in older patients, involve the head and neck, and are often amelanotic with a relatively dense acellular fibrous stroma. They have sarcoma-like tendencies in that they are locally aggressive but are less likely than all other forms of melanoma to spread to regional lymph nodes. A variant of desmoplastic melanoma is desmoplastic neurotropic melanoma (DNM). This variant spreads along nerves leading to local recurrence rates as high as 20% [15]. The treatment of choice is surgical excision with adequate margins. Ironically, there may also be a role for ionizing radiation in the treatment of desmoplastic melanoma despite the relationship between ionizing radiation and cutaneous malignancies [16]. The combination of high risk for local recurrence and low risk for distant metastatic disease in DM/DNM outlines the importance of achieving long-term local control with surgical excision and adjuvant radiotherapy. We chose to use a delayed reconstructive technique in this case until we could confirm a negative margin resection, and we were able to do so because of the patient's health and ability to tolerate a staged procedure. The large size of the keloid in this case predetermined the need for skin graft reconstruction. In patients with unresectable lesions, radiotherapy can be used definitively for local control. With a history of prior irradiation to the site, adjuvant radiation was not an option in this case; thus the patient was treated with surgery only.

In conclusion, we believe that this is the first reported case of a true primary cutaneous melanoma occurring in a keloid. Though ionizing radiation has been linked to increased risk for cutaneous malignancies, it is unclear whether this patient's history of radiation or his extensive life-time sun exposure played a role in the development of this cutaneous melanoma. Clinical vigilance is critical when lesions are not behaving in a typical manner, especially in patients with changing keloids and a history of irradiation to the area. Delayed reconstruction remains a valuable option in situations where positive margins are potentially of concern.
